# A global food plant dataset for wild silkmoths and hawkmoths and its use in documenting polyphagy of their caterpillars (Lepidoptera: Bombycoidea: Saturniidae, Sphingidae)

**DOI:** 10.3897/BDJ.8.e60027

**Published:** 2020-12-10

**Authors:** Liliana Ballesteros Mejia, Pierre Arnal, Winnie Hallwachs, Jean Haxaire, Daniel Janzen, Ian J. Kitching, Rodolphe Rougerie

**Affiliations:** 1 CESAB, Centre de Synthèse et d’Analyse sur la Biodiversité, Montpellier, France CESAB, Centre de Synthèse et d’Analyse sur la Biodiversité Montpellier France; 2 Institut de Systématique, Evolution, Biodiversité (ISYEB), Muséum national d'Histoire naturelle, CNRS,Sorbonne Université, EPHE, Université des Antilles (1st authorship shared between the first two authors), Paris, France Institut de Systématique, Evolution, Biodiversité (ISYEB), Muséum national d'Histoire naturelle, CNRS,Sorbonne Université, EPHE, Université des Antilles (1st authorship shared between the first two authors) Paris France; 3 Ecologie, Systématique and Evolution, Université Paris-Sud, CNRS, AgroParisTech, Université Paris-Saclay, Orsay, France Ecologie, Systématique and Evolution, Université Paris-Sud, CNRS, AgroParisTech, Université Paris-Saclay Orsay France; 4 Department of Biology, University of Pennsylvania, Philadelphia, United States of America Department of Biology, University of Pennsylvania Philadelphia United States of America; 5 Correspondent of Muséum national d'Histoire naturelle, Paris, France Correspondent of Muséum national d'Histoire naturelle Paris France; 6 Associate researcher of Insectarium de Montréal, Quebec, Canada Associate researcher of Insectarium de Montréal Quebec Canada; 7 University of Pennsylvania, Philadelphia, United States of America University of Pennsylvania Philadelphia United States of America; 8 Department of Life Sciences, Natural History Museum, London, United Kingdom Department of Life Sciences, Natural History Museum London United Kingdom

**Keywords:** Lepidoptera, food plant, ecology, life-history traits, caterpillar, polyphagy

## Abstract

**Background:**

Herbivorous insects represent a major fraction of global biodiversity and the relationships they have established with their food plants range from strict specialists to broad generalists. Our knowledge of these relationships is of primary importance to basic (e.g. the study of insect ecology and evolution) and applied biology (e.g. monitoring of pest or invasive species) and yet remains very fragmentary and understudied. In Lepidoptera, caterpillars of families Saturniidae and Sphingidae are rather well known and considered to have adopted contrasting preferences in their use of food plants. The former are regarded as being rather generalist feeders, whereas the latter are more specialist.

**New information:**

To assemble and synthesise the vast amount of existing data on food plants of Lepidoptera families Saturniidae and Sphingidae, we combined three major existing databases to produce a dataset collating more than 26,000 records for 1256 species (25% of all species) in 121 (67%) and 167 (81%) genera of Saturniidae and Sphingidae, respectively. This dataset is used here to document the level of polyphagy of each of these genera using summary statistics, as well as the calculation of a polyphagy score derived from the analysis of Phylogenetic Diversity of the food plants used by the species in each genus.

## Introduction

Herbivorous insects represent a major fraction of global biodiversity (Fiedler 1998) and are central to studies of numerous and diverse ecological and evolutionary processes, such as resource specialisation (Devictor et al. 2008), co-evolution (Thompson 1988) and food web dynamics (Vidal and Murphy 2017). Elucidating the degree of food plant-insect specificity helps understand community assembly, ecosystem dynamics and latitudinal gradients of species richness (Ødegaard 2006). Moreover, insect-plant interactions are central to the understanding of niche breadth and they play a key role in mediating competition that structures communities and backdrop the human view of entire networks of interacting species (Devictor et al. 2008, Forister et al. 2014). The different levels of specialisation observed in phytophagous insects, from strict specialists to highly-generalist species, are traits that are also considered as possibly important drivers of speciation or adaptive radiation (Janz and Nylin 2008, Jousselin and Elias 2019, Wang et al. 2017).

The Lepidoptera families Saturniidae (wild silkmoths) and Sphingidae (hawkmoths, sphinx moths) are amongst the best-known insect families worldwide, both taxonomically and biologically and they are generally characterised by being large-bodied moths (Janzen 1984a). A recently-published taxonomic checklist (Kitching et al. 2018) revealed a combined species richness of around 5000 species globally. These two families exhibit contrasting life-history strategies both as adults - Sphingidae (feeding, long-lived adults) and Saturniidae (non-feeding, short-lived adults) (Janzen 1984a) - and as caterpillars - Sphingidae (fast growing, many toxic plant specialists) and Saturniidae (slow-growing, many tannin and resin-rich plant specialists) (Janzen 1981, Janzen 1984a). In the Neotropics, sphingid caterpillars seem to specialise on only a relatively small number of plant families, feeding on both young and old, relatively tender leaves that contain low molecular weight toxic compounds, whereas saturniid caterpillars feed on tougher, as well as younger, leaves of an often wide range of plant families that contain high levels of large polymeric molecules (tannins, resins) that interfere with digestion (Janzen 1981). Consequently, sphingid caterpillars digest more nutrients per bite and need less time to reach a given full size than do saturniids (Bernays and Janzen 1988).

A massive amount of data is available on the larval food plants in the wild of the two families, both in literature and in institutional and personal databases. For the Lepidoptera as a whole, the HOSTS database (Robinson et al. 2010) comprises the most comprehensive collation of information about what caterpillars overall are believed to eat. It contains some 180,000 records for about 22,000 Lepidoptera species extracted from 1600 documents (Robinson et al. 2010). Although HOSTS has not been updated for almost a decade, the subset of records for the superfamily Bombycoidea has been independently maintained and added to by IJK and this updated version is used here. Another spectacular effort towards gathering food plant data for Lepidoptera is the inventory of caterpillars in the Area de Conservacion Guanascate (ACG) in north-western Costa Rica (Janzen and Hallwachs 2016, Janzen and Hallwachs 2020). It comprises ~ 70,000 records of reared wild-caught larvae of Saturniidae and Sphingidae linked to their DNA barcodes. Besides these two main public data repositories, one of the authors (JH) has built his own personal database for Sphingidae over 20 years, compiling records from literature, web resources, personal field observations and communications from collaborators. In addition, food plant information is also scattered across the published literature, including a few more recent food plant catalogues, such as in Stone (1994), Santin (2004), Meister (2011), but also webpages and personal databases, all of which makes the process of collating and resolving the information very difficult and time consuming.

All three databases cited above are and remain independently maintained and updated. Here we publish a single dataset resulting from their combination. Our aim is to make this massive amount of information available as a single dataset that allows its use for ecological and evolutionary analyses. In particular, we want to investigate the role of food plant use in the evolution of the two families (Arnal et al., in prep.), especially with respect to the degree of polyphagy, defined as the plasticity in the use of different food plants for caterpillars to complete their development. We provide further details about the contents of this dataset in the following sections, as well as a number of caveats to avoid incorrect interpretation and use of these data. In addition to variables summarising the level of polyphagy of the caterpillars of sphingid and saturniid moths, we also provide a polyphagy score, based on a calculation of Phylogenetic Diversity (Faith 1992) of the food plant families used by the species included in the database.

## General description

### Purpose

**The food plant dataset**

This dataset (Suppl. material 1) is a synthesis of current knowledge regarding the food plants eaten by the caterpillars of two families of Lepidoptera (Saturniidae and Sphingidae). It aims to capture the state of knowledge at the time of assembly of the dataset so that it can be used to investigate the role of food plants use breadth in the spatial and temporal evolution of both families (Arnal et al., in prep.).

This dataset of larval food plant records for sphingids and saturniids worldwide is the result of the integration, with significant data reconciliation and standardisation, of these three largely independent data sources:

1) Information for Sphingidae and Saturniidae embedded in the HOSTS database (Robinson et al. 2010); as further added to and refined by IJK, downloaded on 2 March 2018) (hereafter HOSTS);

2) An inventory of the caterpillars, their food plants and parasitoids of Area de Conservacion Guanacaste (ACG, Janzen DH, downloaded on 16 July 2018 for Saturniidae and 18 July 2018 for Sphingidae) (hereafter DHJ);

3) The personal database of Jean Haxaire (Associate Researcher to MNHN, imported on 17 July 2018) (hereafter JH).

A “record” refers to a unique combination of caterpillar species, plant species and source. Records in the dataset resulting from rearing experiments in captivity or from introduced plant species are listed separately as they often do not represent natural insect-plant associations. Redundancy (duplication) of records amongst the three databases following their combination was not a concern for our research objectives; the dataset should be treated as qualitative and the frequency of records ignored (see list of points in next section).

A total of 25,937 records was compiled from the three databases in a single dataset given as Suppl. material 1. Table 1 below provides details of the number of records contributed by each of the independent databases. We followed the plant taxonomy of the International Plant Names Index (IPNI; https://www.ipni.org) and the latest moth taxonomy (Kitching et al. 2018), though both do not coincide with some of the names used by all three sources (see 'call for caution' below).

This compilation provides information for 137 genera and 757 species of Saturniidae and 166 genera and 725 species of Sphingidae.

As an example of the uses of this dataset, we report basic polyphagy variables as well as a polyphagy score, based on the Phylogenetic Diversity (PD, Faith 1992) of the food plants used by the caterpillars of saturniid and sphingid moths. Using a recent dated angiosperm phylogeny (Magallón et al. 2015), we measured the PD score, i.e. the total length of all phylogenetic tree branches connecting the different families of plants eaten by a given moth species *in natura*, using the *pd* function of the *picante* R package (Kembel et al. 2010). The species scores were then averaged within each genus to obtain genus scores in Suppl. material 2. Note that gymnosperm records were excluded from our calculations of PD scores to avoid bias caused by the considerable phylogenetic distance between angiosperms and gymnosperms.

The genus-level polyphagy variables and the polyphagy scores of Saturniidae and Sphingidae genera are provided as Suppl. material 2.

### Additional information

Calls for caution:

1. The correctness of food plant identifications in databases and in literature should be treated with considerable caution, as they were largely made by non-botanists; food plant names used are also subject to taxonomic and nomenclatural uncertainty and their correctness and validity may be considered equivocal in some cases.
2. The previous point also applies to moth names, especially when considering species-level identifications. These may be incorrect or outdated. For example, more than 1500 new species have been described within family Saturniidae in the past decade (Kitching et al. 2018), largely with the support of DNA barcoding analyses. Thus, food plant records may not account for recently split complexes of cryptic species, members of which may have quite different natural histories (e.g. Janzen (2012)).
3. The food plant dataset is derived from known food plant records at the time of its compilation; as such, it represents a snapshot of the knowledge at that time and it may differ from the data compiled in the original sources and then updated independently (e.g. new records and/or corrections (e.g. identification errors or synonymies of the moth/caterpillar or the plant or both)).
4. All records are meant to represent actual instances in which caterpillars were found feeding and developing on the food plant. Records in the DHJ database all result from rearing trials of caterpillars found in the field on the food plant in question and, in many cases, identification of the caterpillar was confirmed through DNA barcoding of the resultant adult moths. A few records, recognised as questionable (e.g. inconsistent locality/identification data) in the HOSTS and JH databases, were filtered out and are not included in the present combined dataset.
5. The food plant dataset does not account for the frequency of use of a given food plant amongst other plants also listed for the same species of moth. The DHJ database, because it is based on individual specimen records, does include quantitative data; however, this information is not incorporated into the combined dataset, although it could bring additional information on local food plant preferences of species and populations. We note that this information would nevertheless be very difficult to analyse and interpret as it is conditional upon the local availability of food plants, as well as possibly seasonal conditions, local variations through time and difficulty of collecting.
6. The previous point also brings a note of caution in that polyphagy, as calculated here from the data available for a given species, may not be translatable to the population or site level and vice versa. A species may have populations in which some caterpillars have a lower level of polyphagy than others, at least in part because the food plants that could be eaten do not occur in that ecosystem and because many species arrive by ecological fitting rather than *in situ* evolution (Janzen 1985a). This is especially the case with species following expanding frontier agriculture into new ecosystems or following contemporary climate changes.
7. Strictly speaking, we define polyphagy as the capacity of a given individual caterpillar to feed and develop (through its complete life cycle) on different food plants. This can only be approximated by considering sibling individuals (as is sometimes the case in the DHJ database), individuals from the same population or, ultimately, from the same species or higher taxonomic categories. We thus acknowledge that the scores of polyphagy at species and genus level should be recognised as human abstractions.
8. Polyphagy is constrained *in situ* by the local availability of food plants - an individual caterpillar cannot be polyphagous on species of plants that are not present.
9. Here we approximated polyphagy scores at species level for saturniid and sphingid moths and we assume that they represent valuable information about the level of plasticity of individuals of the populations of a species to use different food plants. These scores were then used to calculate polyphagy scores at the genus level. Generic level of polyphagy is a human abstraction, but it is seen as relevant information to understand the past diversification dynamics. Plasticity in the use of food plants may have favoured or impeded geographical dispersal and may have mitigated speciation or extinction processes or influenced species' natural histories in many other ways (Janzen 1985b).
10. We acknowledge that the polyphagy level derived from caterpillar plant feeding records approximates, but may not reflect precisely, the plasticity in oviposition site selection by female moths (see, for instance, Janzen 1984b). Indeed, caterpillars may be driven by starvation to feed on a different plant after consuming all leaves of the plant they started to develop on and which had been selected for oviposition by the female.

## Geographic coverage

### Description

The present dataset combines food plant records for saturniid and sphingid species worldwide.

## Taxonomic coverage

### Taxa included

**Table:** 

Rank	Scientific Name	Common Name
family	Saturniidae	Wild silkmoths
family	Sphingidae	Hawkmoths

## Usage licence

### Usage licence

Open Data Commons Attribution License

## Data resources

### Data package title

Global food plant dataset and polyphagy scores for Sphingidae and Saturniidae

### Number of data sets

2

### Data set 1.

#### Data set name

Global food plant dataset for Saturniidae and Sphingidae species

#### Data format

Excel data spreadsheet

#### Number of columns

21

#### Description

Suppl. material 1

**Data set 1.** 

Column label	Column description
Family	Taxonomic family of the moth genus/species
Subfamily	Taxonomic subfamily of the moth genus/species
Tribe	Taxonomic tribe of the moth genus/species
Moth_Genus_name	Genus name
Moth_Species_Name	Species name
Number_PlantGenus	Total number of plant genera known to be eaten in natural environment by caterpillars of this species of moth
Plant_GenusNames	Names of plant genera known to be eaten in natural environment by caterpillars of this species of moth
Number_PlantSpecies	Total number of plant species known to be eaten in natural environments by caterpillars of this species of moth
Plant_SpeciesNames	Names of plant species known to be eaten in natural environments by caterpillars of this species of moth
Number_PlantFamily	Total number of plant families known to be eaten in natural environments by caterpillars of this species of moth
Plant_FamilyNames	Names of plant families known to be eaten in natural environments by caterpillars of this species of moth
Number_PlantOrders	Total number of plant orders known to be eaten in natural environments by caterpillars of this species of moth
Plant_OrderNames	Names of plant orders known to be eaten in natural environments by caterpillars of this species of moth
Number_PlantGenus_Capt	Total number of plant genera known to be eaten in captivity by caterpillars of this species of moth
Plant_GenusNames_Capt	Names of plant genera known to be eaten in captivity by caterpillars of this species of moth
Number_PlantSpecies_Capt	Total number of plant species known to be eaten in captivity by caterpillars of this species of moth
Plant_SpeciesNames_Capt	Names of plant species known to be eaten in captivity by caterpillars of this species of moth
Number_PlantFamily_Capt	Total number of plant families known to be eaten in captivity by caterpillars of this species of moth
Plant_FamilyNames_Capt	Names of plant families known to be eaten in captivity by caterpillars of this species of moth
Number_PlantOrders_Capt	Total number of plant orders known to be eaten in captivity by caterpillars of this species of moth
Plant_OrderNames_Capt	Names of plant orders known to be eaten in captivity by caterpillars of this species of moth

### Data set 2.

#### Data set name

Polyphagy variables and score for Saturniidae and Sphingidae

#### Data format

Excel data spreadsheet

#### Number of columns

13

#### Download URL

​​​​​​​​​​​​​​

#### Description

Suppl. material 2​​​​​​​

**Data set 2.** 

Column label	Column description
Family	Taxonomic family of the moth genus
Subfamily	Taxonomic subfamily of the moth genus
Tribe	Taxonomic tribe of the moth genus
Moth_Genus_Name	Genus name
NumberSampledMothSpecies	Number of moth species within the genus that have food plant information available
TotalMothSpecies	Total number of moth species within the genus
TotalNumberGenus	Total number of plant genera known to be eaten in natural environment by caterpillars of this genus of moth
AverageNumberGenus	The average number of plant genera known to be eaten in natural environments by species within this genus of moth
TotalNumberFamilies	Total number of plant families known to be eaten in natural environments by caterpillars of this genus of moth
AverageNumberFamilies	The average number of plant families known to be eaten in natural environments by species within this genus of moth
TotalNumberOrders	Total number of plant orders known to be eaten in natural environments by caterpillars of this genus of moth
AverageNumberOrders	The average number of plant orders known to be eaten in natural environments by species within this genus of moth
PD_score	Phylogenetic score based on the total branch length in the phylogenetic tree connecting the different families of plant eaten by a given moth species and summarised by genus. NOTE: Only computed from records of plant species eaten in natural environments; records on Gymnosperms were dicarded prior calculation (see text).

## Supplementary Material

9D3525CC-8F4E-5388-87BA-54E30202D975 10.3897/BDJ.8.e60027.suppl1 Supplementary material 1 Food plant dataset for worldwide saturniid and sphingid moths (Lepidoptera, Saturniidae, Sphingidae) Data type food plant and moths association records Brief description This dataset lists food plants known to be fed on by caterpillars of saturniid and sphingid moths, worldwide. It includes both wild and captive records (listed separately). File: oo_467053.xlsx https://binary.pensoft.net/file/467053 Kitching, I.J, Janzen, D.H.J., Hallwachs, W, Haxaire, J

EB690A92-EC26-57F6-BB88-C47D570BFA12 10.3897/BDJ.8.e60027.suppl2 Supplementary material 2 Polyphagy variables and score for Saturniidae and Sphingidae Data type Summary variables, polyphagy score Brief description This table reports summary variables and score of polyphagy for Saturniidae and Sphingidae records of our dataset. Note that gymnosperm records were excluded from our calculations of PD scores to avoid bias caused by the considerable phylogenetic distance between angiosperms and gymnosperms. Polyphagy scores were not calculated for genera feeding on gymnosperms only . File: oo_467055.xlsx https://binary.pensoft.net/file/467055 Ballesteros-Mejia, L., Arnal, P., Kitching, I.J., Rougerie, R.

## Figures and Tables

**Table 1.** General overview of the contribution of each database to our dataset (Suppl. material 1).

Database	Family	Number of records	Geographical coverage
HOSTS	Saturniidae	10586	Worldwide
	Sphingidae	10528	Worldwide
DHJ	Saturniidae	2297	Local in three adjoining ecosystems
	Sphingidae	1322	Local in three adjoining ecosystems
JH	Sphingidae	2401	Worldwide
